# Organic carbon prediction in soil cores using VNIR and MIR techniques in an alpine landscape

**DOI:** 10.1038/s41598-017-02061-z

**Published:** 2017-05-19

**Authors:** Xiaolin Jia, Songchao Chen, Yuanyuan Yang, Lianqing Zhou, Wu Yu, Zhou Shi

**Affiliations:** 1 Institute of Agricultural Remote Sensing & Information Technology Application, College of Environmental and Resource Sciences, Zhejiang University, Hangzhou, Zhejiang 31005 China; 2 Department of Resources and the Environment, XiZang Agriculture and Animal Husbandry College, Linzhi, Tibet 860114 China

## Abstract

Diffuse reflectance spectroscopy (DRS), including visible and near-infrared (VNIR) and mid-infrared (MIR) radiation, is a rapid, accurate and cost-effective technique for estimating soil organic carbon (SOC). We examined 24 soil cores (0–100 cm) from the Sygera Mountains on the Qinghai–Tibet Plateau, considering field-moist intact VNIR, air-dried ground VNIR and air-dried ground MIR spectra at 5-cm intervals. Preprocessed spectra were used to predict the SOC in the soil cores using partial least squares regression (PLSR) and a support vector machine (SVM). The SVM models performed better with three predictors, with the ratio of performance to inter-quartile distance (RPIQ) and *R* ^2^ values typically exceeding 1.74 and 0.73, respectively. The SVM using the DRS technique indicated accurate predictive results of SOC in each core. The RPIQ values of the shrub meadow, forest and total dataset prediction using air-dried ground VNIR were 1.97, 2.68 and 1.99, respectively; the values using field-moist intact VNIR were 1.95, 2.07 and 1.76 and those using air-dried ground MIR were 1.78, 1.96 and 1.74, respectively. We conclude that the DRS technique is an efficient and rapid method for SOC prediction and has the potential for dynamic monitoring of SOC stock density on the Qinghai–Tibet Plateau.

## Introduction

Carbon is an essential component of all organic matter. The carbon cycle and its spatial distribution are intimately involved in the maintenance, development and stability of an ecosystem. The carbon pool in the pedosphere is approximately three times larger than that in the atmosphere^1^. One potential impact of global warming is the acceleration of soil organic carbon (SOC) decomposition and the increase in the release of carbon into the atmosphere^2^. The Qinghai–Tibet Plateau is an important segment of the global terrestrial ecosystem because it is a large and concentrated distribution area of frozen soil at low latitudes. This area is sensitive to global climate change and plays an important role in the Asian climate as well as in global climate change^3^.

Visible and near-infrared (VNIR) and mid-infrared (MIR) radiation measured through diffuse reflectance spectroscopy (DRS) are non-destructive, rapid, accurate and cost-effective techniques for characterizing soil components according to their reflectance. VNIR and MIR were used *in situ* and in the laboratory as an effective alternative for predicting soil properties. The fundamental vibrations of molecules lead to the generation of MIR spectra, and both overtones and combinations collectively lead to the production of VNIR spectra. The difference between the technologies in the physical mechanism determines the essential difference between the characteristics of the two adjacent spectral bands. Morgan *et al*.^4^ summarized the predictive ability of soil organic and inorganic carbon using VNIR DRS from four soil pre-treatments, which used a calibration and validation dataset that was divided according to the intact cores. The soil organic carbon validation had a root mean squared error in prediction (RMSEP) of 5.4 and 4.1 g kg^−1^ for the field-moist and air-dried intact scans, respectively. Li *et al*.^3^ found that the predictive accuracy of SOC from the field spectra was close to that of air-dried conditions, for which the RMSEP were 8.40 and 7.28 g kg^−1^ for the field-moist ground and air-dried intact scans, respectively. Chen *et al*.^5^ compared the prediction accuracy of the PLSR models using VNIR and MIR spectra of air-dried ground arable soil samples. They found that the results using VNIR spectra were generally not as accurate as those using MIR spectra.

The prediction of SOC using VNIR and MIR DRS has been well reported^6–8^. The spectra in the MIR region contain more useful information than the VNIR spectra. In recent years, with the development of science and technology, portable instruments for measuring MIR spectra have gradually emerged, and the price of these machines has decreased. This research is a continuation of the work by Li *et al*.^3^, which evaluated the feasibility of VNIR for predicting SOC in alpine regions and achieved great prediction results. In this research, we investigated the predictive ability in individual soil cores using VNIR and MIR spectra. To our knowledge, no studies have reported the potential to predict SOC in individual soil cores (0–100 cm) in an alpine region because the economic costs of collecting soil material are much higher in such inaccessible regions compared to other areas.

This research focused on the following objectives: 1) to analyse soil spectral characterizations in cores under field-moist intact and air-dried ground conditions using VNIR and MIR spectra; 2) to compare the prediction accuracy using different spectral regions (VNIR and MIR), pre-treatments (field-moist intact and air-dried ground) and modelling approaches; and 3) to quantify the prediction errors of SOC at different depths and in individual soil cores, as well as the difference in organic carbon stock density (SOCD) at depths of 0–30 cm between measured and predicted values.

## Results

### Statistics of soil profile SOC contents

All of the soil samples had a mean SOC concentration of 20.05 g kg^−1^, a median of 15.15 g kg^−1^, and a highly skewed distribution, with a skew parameter of 1.95 (Table 1). The samples provided a wide range for model calibration. Moreover, high variability (according to the values of the coefficient of variation (CV)) indicated that the SOC content was spatially variable within the study area^9^. Soils under shrub meadow exhibited a similar mean to those in forest, despite the median of the former being approximately twice that of the latter. According to the standard deviation (SD) values, forest soils had greater SOC variability than those under shrub meadow.

**Table 1:** Statistics for the concentration of organic carbon under the two land cover types. ^a^Number of soil cores; ^b^Number of all soil samples; ^c^Standard deviation; ^d^Coefficient of variation.

Subset	Unit	N_0_^a^	N_1_^b^	Min	Mean	Median	Max	SD^c^	CV^d^	Skew
Shrub meadow	g kg^−1^	12	155	1.20	20.35	19.45	74.88	14.40	70.76%	1.33
Forest	g kg^−1^	12	175	0.39	19.79	11.41	130.50	23.05	116.47%	1.96
All	g kg^−1^	24	330	0.39	20.05	15.15	130.50	19.44	96.96%	1.95

**Figure 1:**
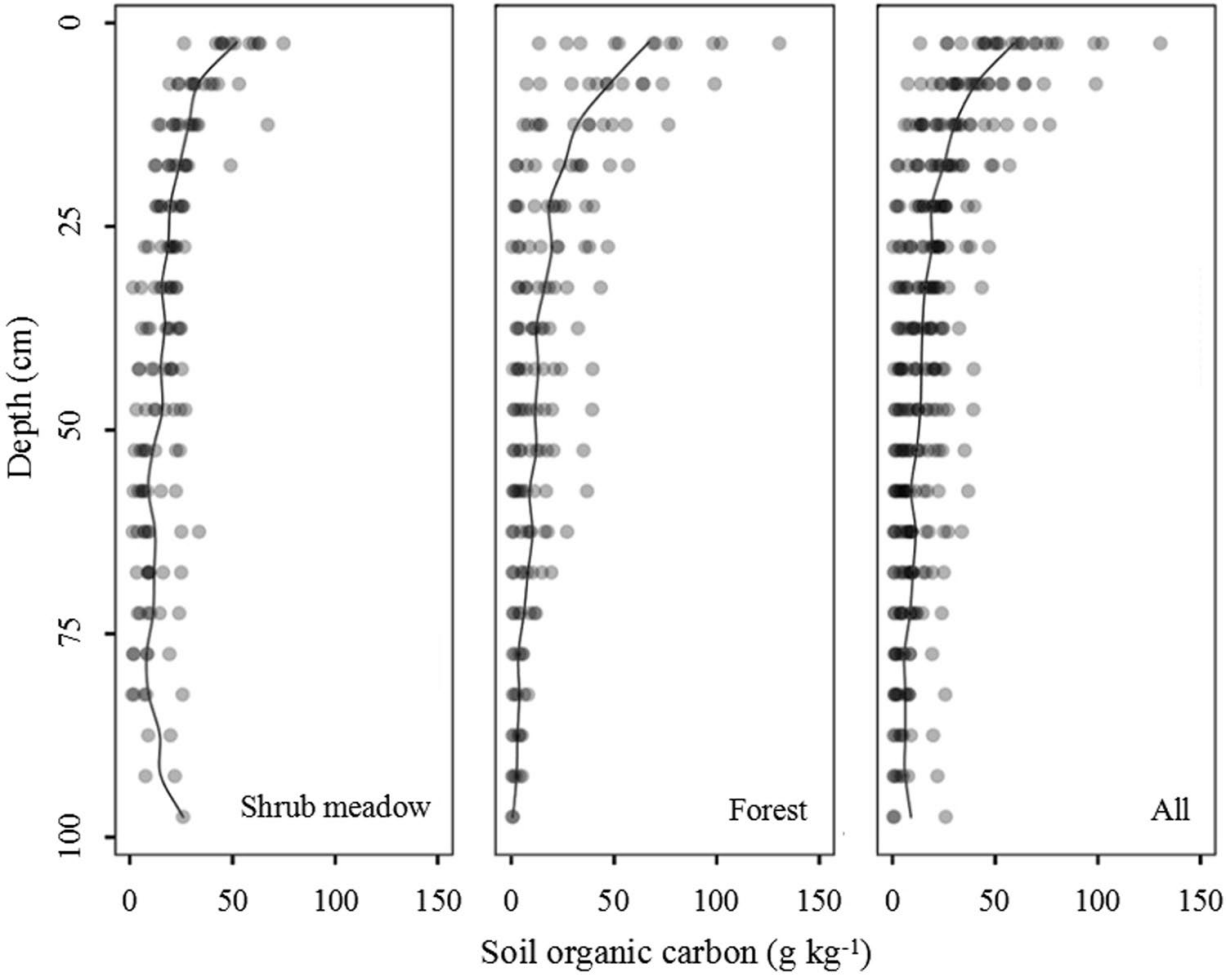
Vertical distributions of soil organic carbon in the soil profiles.

The SOC concentration in forest changed rapidly with soil depth (Fig. 1). The highest SOC concentration in the surface under forest was around twice that under shrub meadow owing to the abundant supply of organic carbon from humus in forests. This also caused a remarkable decrease in the SOC concentration from the topsoil to subsoil in forests. For example, the SOC concentrations between the upper and lower layers were 130.50 g kg^−1^ and 0.39 g kg^−1^ in one representative forest soil profile. In contrast, the SOC concentration under shrub meadow decreased slowly with soil depth. The variability was smaller between the upper and lower layers (e.g., 62.23 and 7.70 g kg^−1^, respectively) in the shrub meadow.

### Qualitative description of the spectral data

**Figure 2:**
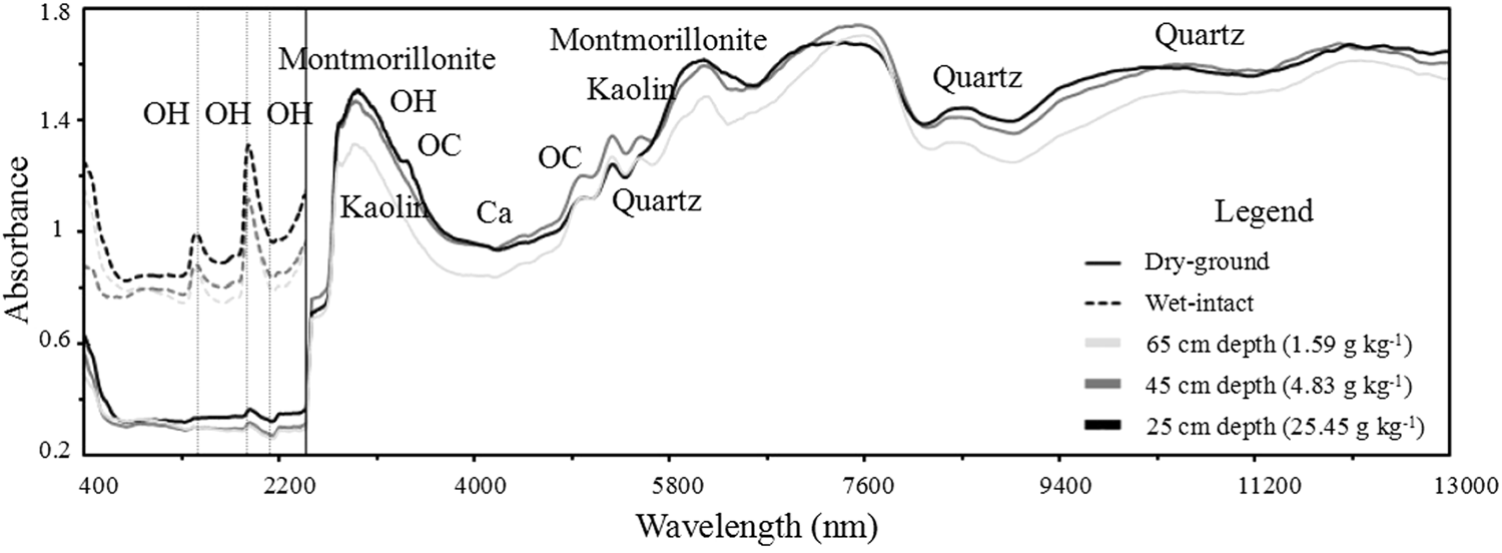
The absorbance spectra at different depths with different contents in one soil core of shrub meadow in the VNIR and MIR regions. OH is the atomic group consisting of oxygen atoms and hydrogen atoms, and OC is the atomic group consisting of oxygen and carbon atoms. The SOC contents in the 65 cm, 45 cm and 25 cm depths were 1.59 g kg^−1^, 4.83 g kg^−1^, 25.45 g kg^−1^, respectively.

Figure 2 provides the absorbance spectra in the VNIR and MIR regions at different depths of one representative soil profile of shrub meadow. The SOC concentrations usually decreased with depth, and the absorbance curves reduced overall with increasing soil depths. A comparison of the field-moist intact and air-dried ground spectra in the range of 400–2450 nm indicated that the soil moisture and structural integrity also affected the soil absorbance spectra; the process of air drying and soil grinding decreased the absorbance spectra and amplitude characteristic peaks. The spectra in the VNIR and MIR regions contained useful information associated with organic carbon, quartz, kaolin and montmorillonite contents (Fig. 2), which were used to establish prediction models of soil properties. The strong absorptions near 1400 and 1900 nm in the VNIR spectra were caused by the O–H functional group of free water, and the absorptions near 2200 nm were caused by the organic matter^10, 11^. In the MIR region, the spectra information mainly responded to mineral properties, such as quartz, kaolin and montmorillonite.

### Performance of the prediction models

In all prediction models, the best results with different pre-treatments are shown in Table 2, and the Haar wavelet transform plus first derivative performed better than the other pre-treatments. Therefore, the Haar wavelet transform plus first derivative was effective for modelling SOC-related information from the soil spectra. The PLSR results exhibited average values of RMSEP and *R* ^2^ of 12.41 g kg^−1^ and 0.70, respectively. The SVM regression models (average values of RMSEP and *R* ^2^ of 8.31 g kg^−1^ and 0.84) performed better in predicting the SOC concentration under different land cover types than the PLSR models. Furthermore, the prediction results were best for the forest soil, with RMSEP and *R* ^2^ values of 7.26 g kg^−1^ and 0.92, respectively, of which the spectra data were pre-processed by the Haar wavelet transform plus the first derivative method.

**Table 2:** Performance of the best spectroscopic models using the validation set. ^a^Spectral transformation (Haar = Haar wavelet transform; FD = first derivative; SNV = standard normal variate; MSC = multiplicative scatter correction); ^b^Multivariate calibration model; ^c^Predictor used in the models (VNIR_w_ = field-moist intact VNIR spectroscopy; VNIR_d_ = air-dried ground VNIR spectroscopy; MIR = air-dried ground MIR spectroscopy); ^d^Root mean square error of prediction (g kg^−1^).

Subset	Treatment^a^	MVC^b^	Predictor^c^	RMSEP^d^	*R* ^2^
Shrub meadow	Haar	SVM	VNIR_w_	7.09	0.82
	SNV + FD	PLSR	VNIR_w_	9.65	0.77
	Haar + FD	SVM	VNIR_d_	7.00	0.83
	Haar + FD	PLSR	VNIR_d_	8.84	0.82
	Haar + MSC	SVM	MIR	7.79	0.73
	Haar + MSC + FD	PLSR	MIR	9.55	0.63
Forest	Haar + FD	SVM	VNIR_w_	9.39	0.87
	MSC + FD	PLSR	VNIR_w_	14.36	0.76
	Haar + FD	SVM	VNIR_d_	7.26	0.92
	Haar	PLSR	VNIR_d_	14.38	0.75
	Haar + SNV + FD	SVM	MIR	9.93	0.89
	Haar	PLSR	MIR	16.77	0.59
All	Haar	SVM	VNIR_w_	9.12	0.81
	SNV + FD	PLSR	VNIR_w_	12.22	0.67
	Haar + FD	SVM	VNIR_d_	8.06	0.86
	SNV + FD	PLSR	VNIR_d_	11.86	0.72
	Haar + SNV + FD	SVM	MIR	9.19	0.82
	Haar + SNV + FD	PLSR	MIR	14.06	0.56

**Figure 3:**
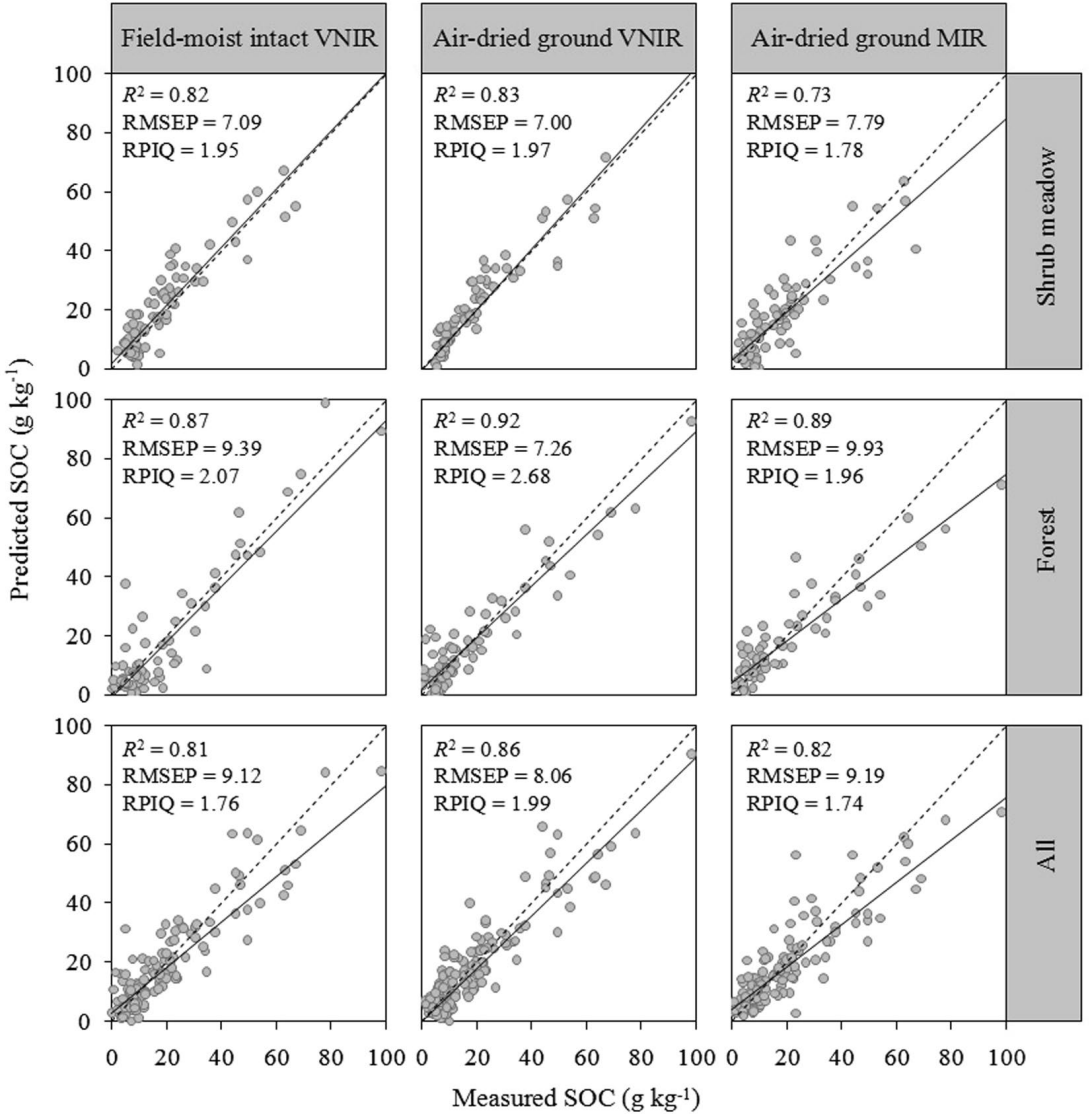
Predicted SOC plotted against measured SOC in validation sets using SVM models.

The ratio of performance to inter-quartile distance (RPIQ) values from the SVM models for the VNIR and MIR predictors were generally over 1.70. Although the MIR spectroscopy was able to estimate the SOC contents in profiles with high RPIQ values, the fitted regression line in the models of air-dried ground MIR was clearly under the 1:1 line (Fig. 3), indicating that the SOC content values were significantly underestimated at high concentrations. In summary, the VNIR calibration models exhibited better quantitative prediction abilities than the MIR models in the soil profiles, especially using the air-dried ground VNIR. According to research by Kuang and Mouazen^12^, the variability in the range of SOC content is a crucial factor influencing the accuracy of calibration models, and the results revealed that a larger SD and wider range explained the variability of SOC content and led to a larger *R* ^2^ and RPD but also a larger RMSEP. Since the land cover types were different and the SOC concentration in forest had a wider distribution than that in shrub meadow, the prediction of forest soil had the highest prediction accuracy, followed by the total dataset and finally the shrub meadow subset.

### The prediction accuracy in different soil core depths

**Figure 4:**
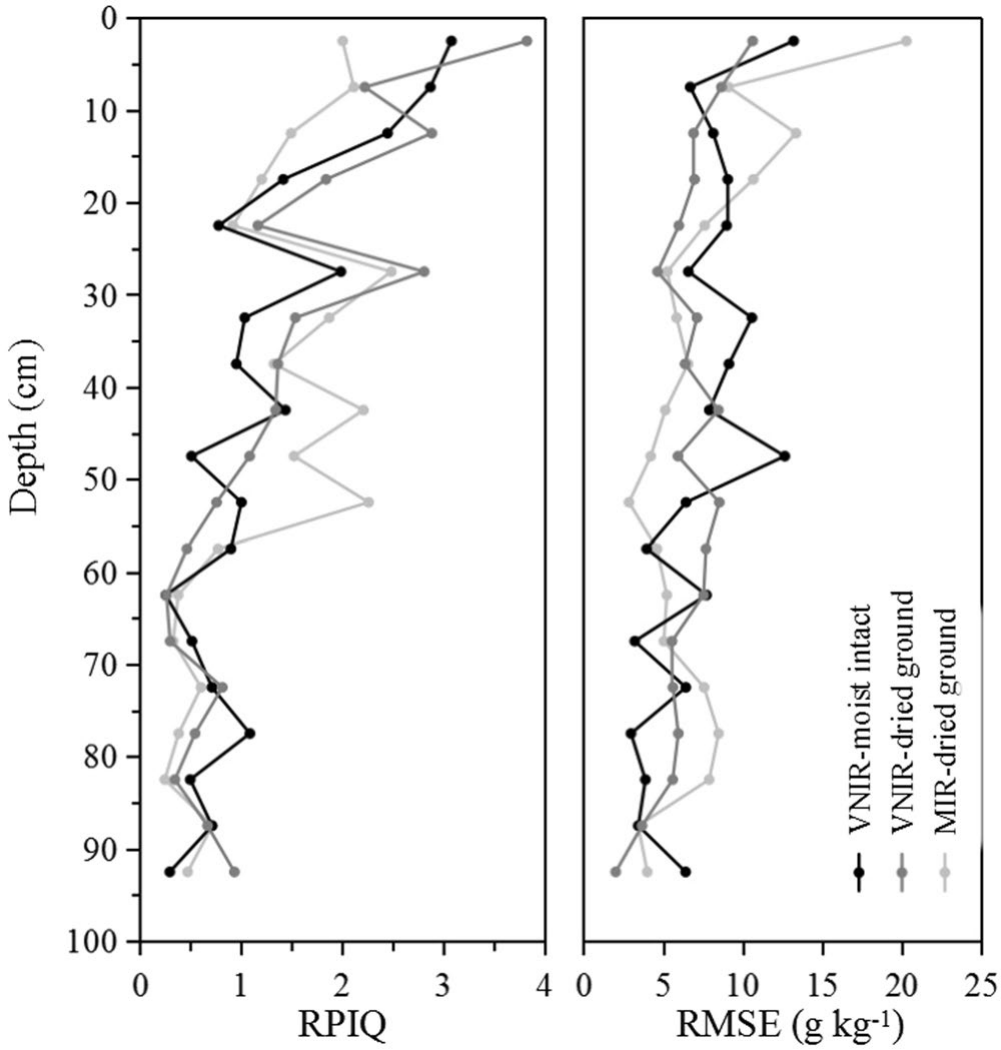
VNIR results from moist intact soil and VNIR and MIR results from dried ground soil at different depths in the soil profiles with the validation sets of total samples by SVM models.

The RMSEP of SVM models under all land cover types indicated a descending trend as soil depths increased (Fig. 4). Between soil depths of 0 and 30 cm, with a high RPIQ and low RMSEP, the MIR results generally had lower predictive accuracy of SOC concentration than the models of VNIR, and the air-dried ground VNIR had the highest accuracy. MIR performed best at soil depths between 30 and 60 cm, gradually increased to a small peak and then decreased rapidly. Below 60 cm soil depth, the best model was the field-moist intact VNIR followed by the air-dried ground VNIR and MIR. In this range, the accuracy was low but it had little influence on the prediction of SOCD per 1 m depth because of its low SOC concentrations as well as low contribution to whole profiles (Fig. 1). The variability in the calibration set affected the accuracy of the prediction models. A larger SD and wider range of the property dataset resulted in larger *R* ^2^ and RPD values but also larger RMSE values^12^. Due to the small concentration ranges in deep soils compared to topsoil, the RPIQ and RMSE values decreased with depth. However, these models were accepted without the RMSE values increasing the measurement error above a desired threshold.

Every soil core had high prediction accuracy; furthermore, the average PRIQ values of the three predictors, including the field-moist intact VNIR, air-dried ground VNIR and air-dried ground MIR spectra, were 2.17, 2.43, and 1.85, respectively. The *R* ^2^ values of all of the soil cores in the field-moist intact VNIR, air-dried ground VNIR and air-dried ground MIR models were greater than 0.70 (the average *R* ^2^ values were 0.86, 0.92, 0.81) with an RMSEP average value of 7.81, 6.80 and 8.96 g kg^−1^. The values of RPIQ, *R* ^2^ and RMSEP indicated that the soil cores could be predicted well, whereas the accuracy for each core varied. The results obtained from the air-dried ground and field-moist intact VNIR spectra were relatively more accurate; the RPIQ and *R* ^2^ values commonly exceeded 2.00 and 0.80. The prediction accuracy of the individual core in MIR models was comparatively lower. The prediction results for forest soils were slightly better, with average RPIQ values of 2.47, 2.82 and 1.74 for the field-moist intact VNIR, air-dried ground VNIR and air-dried ground MIR spectra, respectively, compared with those under shrub meadow (average RPIQ values of 1.87, 2.04 and 1.95, respectively). In general, the models performed best using air-dried ground VNIR spectra in forest. Table 3 shows the predicted results using the SVM regression models.

**Table 3:** Predicted SOC content compared with measured SOC content in each core of the validation sets using SVM. ^a^Predictor used in the models (VNIR_w_ = field-moist intact VNIR spectroscopy; VNIR_d_ = air-dried ground VNIR spectroscopy; MIR = air-dried ground MIR spectroscopy); ^b^‘S_V_’ is the soil cores of validation dataset in the shrub meadow; ^c^‘F_V_’ is the soil cores of validation dataset in the forest; ^d^the unit of RMSEP is g kg^−1^.

Predictor^a^	Index	S_V1_^b^	S_V2_	S_V3_	S_V4_	S_V5_	F_V1_^c^	F_V2_	F_V3_	F_V4_	F_V5_
VNIR_w_	RMSEP^**d**^ (g kg^−1^)	5.23	9.22	7.02	5.80	6.53	5.83	5.14	12.79	10.81	9.70
	*R* ^2^	0.90	0.87	0.77	0.88	0.72	0.98	0.92	0.80	0.87	0.89
	RPIQ	2.50	2.43	1.48	1.18	1.75	6.04	1.50	1.66	1.90	1.28
VNIR_d_	RMSEP (g kg^−1^)	4.87	10.94	5.98	5.94	4.43	5.91	5.99	10.24	8.13	5.54
	*R* ^2^	0.96	0.86	0.84	0.96	0.89	0.96	0.91	0.91	0.97	0.98
	RPIQ	2.69	2.05	1.74	1.15	2.57	5.96	1.28	2.07	2.52	2.25
MIR	RMSEP (g kg^−1^)	9.73	8.56	3.53	6.48	7.72	11.70	10.77	9.25	16.83	4.99
	*R* ^2^	0.80	0.71	0.95	0.69	0.56	0.83	0.82	0.93	0.88	0.97
	RPIQ	2.30	1.21	3.71	1.05	1.48	1.81	1.90	1.35	2.09	1.54

### Prediction of soil organic carbon stock density

The mean soil bulk density (BD) under shrub meadow and forest was 1.02 and 1.17 g cm^−3^, respectively. This paper analysed the soil texture of the samples and found that the content proportions of sand, silt and clay were 54.06–65.30%, 15.50–25.68% and 10.83–20.26%. The soil texture was mainly classified as sandy loam according to the United States Department of Agriculture (USDA) texture classification system. The formula of the SOCD (g cm^−2^) is as follows^13^:

1 $$SOCD=BD\times h\times \sum _{i=1}^{n}SO{C}_{i}$$

where *BD* is the mean soil bulk density (g cm^−3^), *SOC*_*i*_ is the concentration of SOC each increment (g kg^−1^) and *h* is the thickness of the soil increment (cm).

**Figure 5:**
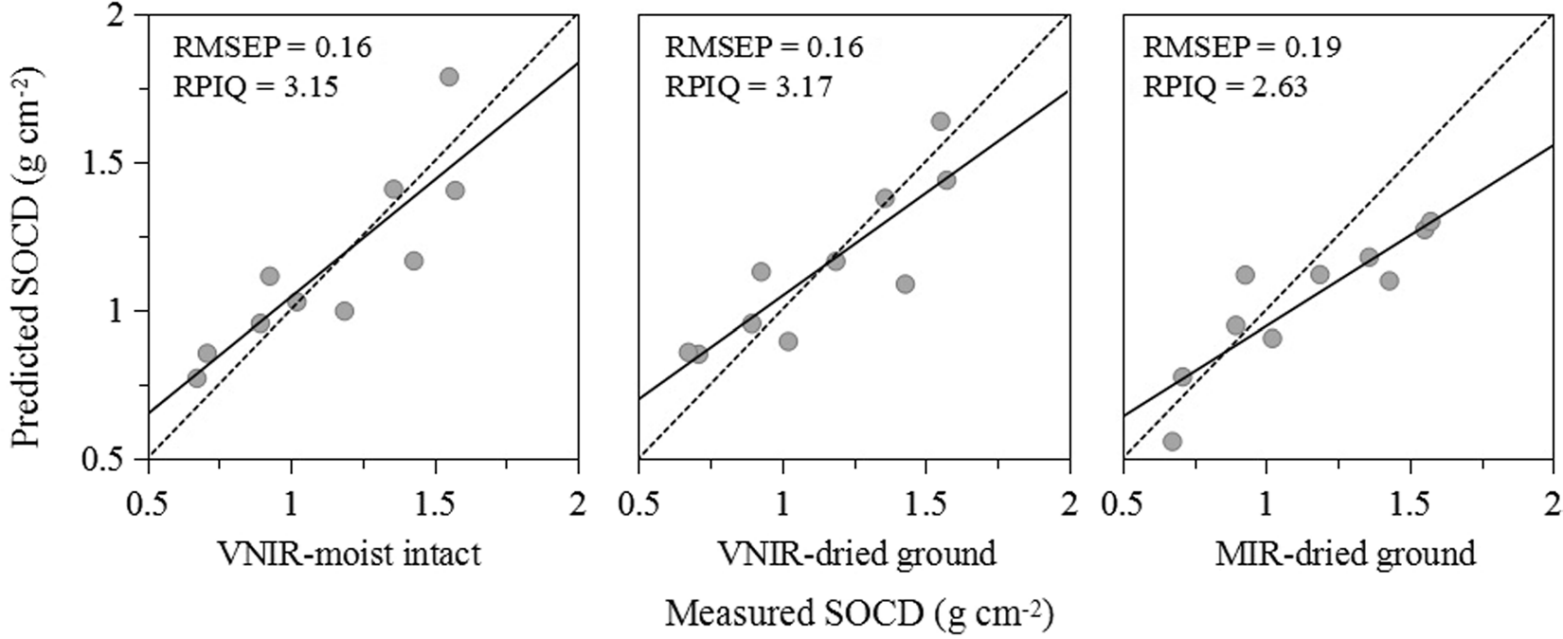
The values of predicted SOCD compared with measured SOCD using the different spectral methods.

As shown in Fig. 1, the SOC content in the topsoil (0–30 cm) was the primary component of the SOCD in the whole soil layer. The SOCD in the 0–30 cm layer under shrub meadow was 72.20 percent of that in the 0–100 cm layer, and the proportion was 80.85 percent under forest. The average content of SOCD measured in the cores was 1.13 g cm^−2^ in the total dataset using SVM models, compared with that predicted by SVM using field-moist intact VNIR, air-dried ground VNIR, air-dried ground MIR (1.14, 1.14 and 1.03 g cm^−2^, respectively). The average SOC content predicted by the air-dried ground VNIR was most similar to the measured SOC content, and the models using air-dried ground VNIR had the best results in an individual core followed by field-moist intact VNIR and air-dried ground MIR (Fig. 5). The accuracy results of SOC and SOCD were identically predicted by different spectra. The model accuracy with RPIQ values above 2.0 exhibited the quantitative ability of three pre-treatments, whereas soil samples with high SOCD were underestimated to different degrees.

## Discussion

In all models, the dried ground MIR models obtained the lowest accuracy, and the dried ground VNIR models provided the best results, followed by the moist intact VNIR models, for SOC and SOCD. Through the establishment of PLSR models, Chen *et al*.^5, 9^ compared the prediction accuracy between VNIR and MIR spectra of air-dried ground arable soil samples and found that the results using MIR spectra were more accurate than those using VNIR spectra. However, we divided the calibration and validation datasets into a complete soil core in our study, whereas Chen *et al*. only studied topsoil samples. Moreover, differences in land cover and ecosystem types may also cause uncertainty in the modelling. The SVM models of the air-dried ground soils using VNIR spectra were evaluated to determine the optimal results because the air-dried ground soil was maximized to weaken the influence of moisture. The SVM models using field-moist intact VNIR spectra predicted values with lower accuracy than those using the air-dried ground soils, but the difference between them was not substantial. The SVM models using MIR spectra had the worst ability to predict the SOC content due to seriously underestimating the high SOC concentration.

The SVM models using different spectra were evaluated to determine the optimal results for SOC prediction under forest, then under shrub meadow. Furthermore, the variability in the SOC concentration had a great influence on the accuracy of the prediction models. Kuang *et al*.^12^ also reached the same conclusion. A wider range in the property dataset resulted in larger *R* ^2^ and RPD values but also larger RMSE values. Because of the small concentration ranges in deep soils compared to topsoil, the RPIQ and RMSE values decreased with depth in the soil cores. These models would be acceptable if the RMSE values did not increase the measurement error above a desired threshold. With the RPIQ values of different models in the paper, we conclude that it was necessary that the natural land covers were divided into different types (shrub meadow and forest) for prediction. In the field, the difference between shrub meadow and forest was obvious and easily distinguished. If there is a requirement for high accuracy prediction, it is necessary to undertake separate modelling and this is easy to implement.

Many studies^14–16^ took soil samples of different depths from the same soil profile as independent individuals, which led to the samples from the same profile being divided into both calibration and validation datasets. However, in practical application, it is not logical to allocate data in this manner; instead, samples from the same profile should be divided into the same datasets, as shown in the current study. Our results indicate that VNIR and MIR DRS is an effective technique for rapidly estimating the SOC concentration in soil cores on the Qinghai–Tibet Plateau and that the models have the potential to estimate the SOC content at different depths. The results *in situ* using VNIR spectra were not as accurate as those obtained in the laboratory. In the future, we plan to build a data library of SOC prediction in Tibet. We can increase the prediction accuracy through transfer methods, such as direct standardization (DS), piecewise direct standardization (PDS), external parameter orthogonalization (EPO) and so on. These algorithms were developed to transfer spectra measured in one way so that it would seem they were measured in another way^17^. The field-moist intact spectra can be observed as approximating the air-dried ground spectra using these methods; then, we can achieve the results *in situ* as accurately as those in the laboratory.

This paper provides a method with which to predict SOC content in the Qinghai–Tibet Plateau that uses intact soil cores for predicting the SOC content throughout soil cores and for quantifying the SOC variation at different depths. One of the limitations of spectral scanning and establishing SOC models is soil variability and heterogeneity. Therefore, in future research, we should sample more soil cores to enlarge the calibration dataset, and correspondingly build a spectral library categorized by a local ecological environment for use in the prediction of SOCD across the whole Qinghai–Tibet Plateau.

## Materials and Methods

### Soil sampling

**Figure 6:**
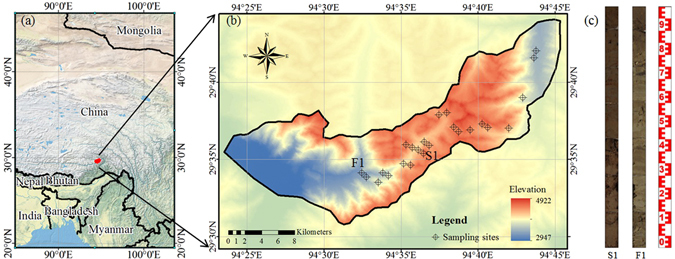
Location of the study area (**a**), soil core sites (**b**) and representative soil cores (**c**). S1 was the soil core in the shrub meadow and F1 in the forest. The map was created using ArcGIS 10.2 (Environmental Systems Research Institute Inc. Redlands, California, USA), of which web set was http://www.esri.com/software/arcgis.

The study area is located in the Sygera Mountains on the southeastern Qinghai–Tibet Plateau of China (Fig. 6a). It covers approximately 305 km^2^ between 29.50°N and 29.75°N and 94.42°E and 94.75°E. The elevation of the forestland ranges from 3300 to 4300 m above sea level, and the alpine shrub and meadows are found above 4300 m. The average annual precipitation is 676 mm, with most rainfall occurring in summer, and the mean annual temperature is 15.8 °C. The soil belongs to four different classes (Cambisols, Luvisols, Phaeozems and Umbrisols) according to the World Reference Base soil classification system. The soil texture in the study field can mainly be classified as sandy loam, sandy clay loam and clay loam according to the USDA texture classification system. The content proportions of sand, silt and clay were 39.50–76.30%, 15.50–30.50% and 7.83–30.00%, respectively.

Twenty-four cylindrical soil cores (5 cm diameter) were collected to a maximum depth of 1 m or to the depth of a sampling-restrictive horizon (Fig. 6c). The soil cores were sampled in August of 2013 and 2014 from the foot of the mountains (approximately 2900 to 4500 m altitude) along the road.

### *In-situ* spectral measurement

An Analytical Spectral Devices FieldSpec Pro FR spectrometer (Analytical Spectral Devices Inc., Boulder, USA) was used to measure the spectra reflected from the field-moist intact soil, with a high-intensity contact probe and an independent light source for the soil cores. The instrument has a spectral range of 350–2500 nm and a resolution of 3 nm at 700 nm and 10 nm at 1400 and 2100 nm. The spectra have a sampling resolution of 1 nm. A panel with 99% reflectance was used as a white reference before each measurement.

All of the soil cores were prepared for the first scan by cutting the soil column in half lengthwise. The probe was placed on the cut, flat, surface of each sample half, and the reflectance spectra was measured every 5 cm from the top to the bottom of each core. Each scan collected 10 spectra, the average of which was recorded as the mean diffuse soil reflectance spectra. In total, 330 spectral curves were collected for 24 soil cores.

### Laboratory measurement

Each depth increment of soil samples was air-dried, ground, passed through a 2-mm sieve, and placed in a petri dish of 1.5 cm depth and 10 cm in diameter. The reflectance spectra of air-dried ground soil samples were measured using the ASD FieldSpec Pro FR spectrometer (the same instrument used for scanning the field-moist intact soil). Then, the air-dried ground spectra were scanned with a 4100 ExoScan FTIR spectrometer (Agilent Technologies Inc., California, USA). The MIR instrument has a spectral range of 650 to 4000 cm^−1^ and a resolution of 4 cm^−1^.

In summary, there were three scanning types, field-moist intact VNIR, air-dried ground VNIR, and air-dried intact MIR. The soil samples were scanned in the laboratory under the same ambient conditions that imitated the outdoor conditions of sampling in Tibet: 15 °C and 12% relative humidity. The three spectra were recorded using contact-probe assemblies that touched the surface of the samples^18^. The major difference between field-moist intact VNIR and air-dried ground VNIR spectra was the preprocessing of soil samples, and the other conditions were almost the same. Finally, 330 spectra were obtained for each scanning type.

The SOC concentrations in the soil cores were measured to correspond to spectra in each depth increment. First, 0.1 mol L^−1^ hydrochloric acid was used to remove soil inorganic carbon; then, the concentration of organic carbon was determined using the dry combustion method at 1100 °C with a multi N/C 3100 instrument (Analytik Jena AG, Germany)^19^. The BD was measured using the core method. This method uses steel cylinders of known volume (100 ml, 400 ml) that are driven into the soil vertically or horizontally by percussion.

### Spectral preprocessing

Because of the instrumental noise in the spectral range from 350–399 nm and from 2451–2500 nm, the VNIR spectral data from 400–2450 nm were retained for further analysis. To obtain the linear relationship between the spectra and the SOC concentration, we converted the VNIR and MIR reflectance spectra to absorbance spectra using the log(1/*R*) transformation, where *R* is the reflectance spectra. Several mathematical pre-treatments on the spectra were applied to remove physical variability and enhance features of interest^20^. The preprocessing included the Haar wavelet transform, Savitzky–Golay smoothing and the first derivative technology^21, 22^, and the best predictive result in all models by different pre-processing methods was recorded. Haar wavelet transform can be compared to a set of image filters. The signals are input to a high-pass filter and a low-pass filter to achieve a high frequency component (detail information) and low frequency component (approximate information). In brief, the Haar wavelet transform is a novel, no-multiplier structure used to lessen the computational complexity.

### Spectroscopic modelling and statistical analyses

The soil cores were defined as forest and shrub meadows based on the natural land cover. According to the elevation, two thirds of the soil cores in each land cover class were divided into a calibration set, and the remaining cores were divided into a validation set. Among the cores under shrub meadow, seven cores were used for calibration and the remainder for validation. The same approach was used for data partitioning of forest and for the total dataset. The calibration set was used to train the models, and the validation set was used to evaluate the performance of the models individually.

After spectral pre-treatments, PLSR and SVM were used to predict the SOC concentration. When there are many predictor variables that are highly collinear, PLSR aims at predicting one dataset from the other sets using a linear multivariate model. This technique is closely related to principal components regression (PCR), but outperforms PCR. The PLSR can realize the functions of data structure simplification, variable correlation analysis and regression modelling synchronously^7^. The SVM is based on a kernel function that causes the linearly inseparable problem in the original space to be transformed into a linearly separable problem in the feature space^23^. We used a radial basis function kernel, which is a typical general-purpose kernel. Compared with linear models, it not only does not increase the computational complexity but also avoids the “curse of dimensionality” to some extent. The accuracy of the models was assessed using three indices: RMSEP, *R* ^2^ and RPIQ. When the value of *R* ^2^ and RPIQ increase and the value of RMSEP decrease, the accuracy of a model is considered to increase.
